# Delirium Following Concomitant Use of Clozapine and Tetrabenazine in Treatment-Resistant Schizophrenia

**DOI:** 10.1192/bjo.2026.11782

**Published:** 2026-06-30

**Authors:** Divyang Golani, Swapnil Aloney, Anik Pal, Ragini Patil

**Affiliations:** Datta Meghe Institute of Higher Education and Research, Wardha, India

## Abstract

**Aims:**

A 38-year-old male with an 8-year history of schizophrenia presented with inadequate response to multiple antipsychotics and tardive dyskinesia. Tetrabenazine was initiated for dyskinesia, and Clozapine was started with gradual titration. On day 14, T. Clozapine was increased from 125 mg to 150 mg. Following this, the patient had an episode of sleep disturbances and purposeless motor movements that lasted for half an hour. Afterwards, the patient slept well, and the following day, his mental status examination revealed no new findings. The following night, 3 hours after receiving the second dose of Clozapine 150 mg, the patient developed disorientation, restlessness, and visual hallucinations. He was not cooperative with any instructions. Laboratory investigations, including haematological and metabolic parameters, were performed and were within normal limits. The acute onset of these clinical features supported the diagnosis of Clozapine-Induced Delirium.

**Methods:**

Case Report

**Results:**

Management included immediate discontinuation of all medications. Pharmacological sedation with parental Lorazepam and Haloperidol was required. Even after this, the patient had to be retrained to start intravenous Ringer’s lactate for maintenance of hydration. Delirium subsided within 18 hours, and orientation returned without any residual deficits. S. Clozapine levels could not be done due to financial limitations. This adverse drug reaction suggests a probable link between Clozapine and Tetrabenazine, precipitating delirium, possibly through cholinergic-dopaminergic imbalance.

**Conclusion:**

To the best of our knowledge, this is the first known case of its kind where the combination of Tetrabenazine with Clozapine, at relatively low dosage, has led to the life-threatening side effect of delirium. This case highlights the complexities of treating a patient who has become hypersensitive to both first-generation and second-generation antipsychotics, including Clozapine. While further research needs to be done regarding this unusual reaction, clinicians should remain vigilant for delirium during clozapine initiation, particularly when used alongside VMAT2 inhibitors such as tetrabenazine.

